# Association between alcohol dehydrogenase-2 gene polymorphism and esophageal cancer risk: a meta-analysis

**DOI:** 10.1186/s12957-016-0937-y

**Published:** 2016-07-22

**Authors:** Ning Mao, Siyao Nie, Bin Hong, Chao Li, Xueyuan Shen, Tao Xiong

**Affiliations:** Department of Cardiothoracic Surgery, Yongchuan Hospital of Chongqing Medical University, No. 439 Xuanhua Road, Yongchuan District, Chongqing, 402160 China; Department of Infectious Disease, Yongchuan Hospital of Chongqing Medical University, Chongqing, 402160 China

**Keywords:** Esophageal cancer, Alcohol dehydrogenase 1B, Polymorphism, Meta-analysis

## Abstract

**Background:**

It has been shown that gene polymorphisms may play an important role in the carcinogenesis of esophageal cancer. This study is to investigate the role of alcohol dehydrogenase 1B (ADH1B) gene Arg47His polymorphism in esophageal cancer susceptibility.

**Methods:**

Case-control studies published between January 2000 and June 2015 were searched to retrieve relevant articles. The pooled odds ratio (OR) and 95 % confidence interval (CI) were employed to calculate the strength of association.

**Results:**

A total of 23 relevant articles were finally selected for the analysis, including 9338 esophageal cancer patients and 14,896 matched controls. Overall, we found that the 47His allele was significant associated with the decreased risk of esophageal cancer when compared with the 47Arg allele in total populations (A vs. G: OR = 0.67, 95 % CI = 0.59–0.76, *P* < 0.00001). This protective relationship was observed under other genetic models as well (*P* < 0.00001). Subgroup analysis by ethnicity showed that ADH1B Arg47His variant was associated with the decreased esophageal cancer risk under all the genetic models (*P* < 0.00001) among Asians, especially in Chinese and Japanese; while in non-Asians, no significant correlation was detected in any genetic models (*P* > 0.05). Furthermore, Arg/Arg genotype of ADH1B Arg47His variant combined with drinking, smoking and males appeared to show a high risk in patients with esophageal cancer.

**Conclusions:**

Our results suggested that ADH1B gene Arg47His variant was associated with the decreased esophageal cancer risk. Genetic-environmental interaction should be further considered in the future researches.

## Background

Esophageal cancer (or oesophageal cancer), a type of cancer arising from the esophagus, is the eighth most common cancer and the sixth leading cause of cancer-related death worldwide [1]. Squamous cell carcinomas (SCC), which is more common in the developing world, and adenocarcinomas (AC), which is more common in the developed world, are two main forms of histologically confirmed esophageal cancer [2, 3]. The clinical symptoms include difficulty in swallowing, enlarged lymph glands around the collarbone, a dry cough, weight loss, and possibly hematemesis [4]. The established risk factors for this disease are environmental factors (alcohol drinking, smoking, infecting bacteria or virus), genetic factors (mutations in enzymes that metabolize alcohol), cultural factors (high-temperature food items such as pork braised in brown sauce and old stocked rice), obesity and gastroesophageal reflux [5–8]. The incidence of esophageal cancer is threefold higher in men than women [9] and is high in east Asia, southern Africa, and southern Europe while is low in North America and other parts of Europe [10]. The highest reported incidence and mortality rates occur in China, which is almost 20 to 30 times higher than that in the USA [11]. According to cancer statistics, there will be an estimated 16,980 new cases and 15,590 deaths in both sexes in the USA in 2015 [12]. Although many advances in diagnosis and treatment such as endoscopic resection, chemotherapy, and surgery have the potential to substantially reduce mortality and morbidity [13, 14], the prognosis of patients with esophageal cancer remains poor and the 5-year survival rate is still low, ranging from 15 to 25 % [15, 16]. Therefore, identifying new biomarkers for early diagnosis and treatment is vital to decide therapeutic options, improve treatment efficiency, and predict prognosis [17].

Evidences have shown that gene polymorphisms may play an important role in the carcinogenesis of esophageal cancer [18]. Alcohol dehydrogenase gene (ADH), at chromosome 4, is a key cytosolic enzyme for ethanol [19]. It encodes at least seven ADH isoforms (ADH1–ADH7), each with slightly different properties [20], and may be involved in the metabolic pathways of several neurotransmitters [21]. The ADH1B (also known as ADH2) gene is located on human chromosome 4q21-q23. It is the locus responsible for most of the ADH activity on ethanol in the liver [22]. Single nucleotide polymorphisms (SNPs) occurring in this gene may be capable of altering ethanol metabolism, and individuals expressing the ADH1B variants would have different alcohol elimination rates [23]. One of the most studied SNP was Arg47His (rs1229984), a G to A base transition in exon 3 leading to the substitution of arginine (ADH1B*1) to histidine (ADH1B*2) at codon 47th position. ADH1B*2 is common in more than 90 % of Asians and reduced their risk for alcoholism but fewer than 20 % of Caucasians or Africans [24]. This variant was shown to be strongly associated with alcohol dependence, abuse, consumption, and alcohol-induced liver diseases [25, 26].

Several studies have identified the relationship between ADH1B polymorphism and esophageal cancer susceptibility, but the consistent results were not obtained. For example, Ito et al. demonstrated that ADH1B Arg47His variant was associated with esophageal cancer in Japanese and might be used in personalized prevention programs [27], while Ma et al. did not show significant associations between variations in the ADH1B gene and esophageal squamous cell carcinoma (ESCC) risk in Chinese [28]. Although previous meta-analyses were performed to evaluate this association [29–31], limited articles were selected, only Asian population was used for analysis, and some repeated participants were included. Furthermore, Asians and Caucasians may have different incidence, distribution, and susceptibilities to esophageal cancer due to different heritage backgrounds [32]. All these factors may influence the reliable of the results. Therefore, we conducted the present meta-analysis to review all the published articles among any ethnicities to obtain a relative reliable result.

## Methods

### Identification of relevant studies

Electronic databases of PubMed, Web of Science, Medline, Embase, CNKI (China National Knowledge Internet), and Wanfang were comprehensively searched to retrieve relevant articles published between January 2000 and June 2015. The MeSH terms were as follows: “esophageal cancer or oesophageal cancer or esophageal squamous cell carcinomas or esophageal adenocarcinomas,” “alcohol dehydrogenase or ADH1B or ADH2,” and “polymorphism or variant or mutation” as well as their combinations. The equivalent Chinese characters were used in Chinese database. We also manually searched the references of included studies in order to obtain more related articles. Our analysis only considered studies that were written in English and Chinese.

### Inclusion and exclusion criteria

The eligible articles must meet the following criteria. The inclusion criteria were as follows: (1) case-control studies evaluating the correlation of ADH1B Arg47His polymorphism in esophageal cancer occurrence; (2) patents with esophageal cancer should be diagnosed clinically and confirmed histologically, the controls should be age-, ethnicity-matched participants without malignancy and digestive and chronic diseases; (3) the genotype information were available to extract; and (4) the genotypes of controls were consistent with Hardy-Weinberg equilibrium (HWE). The exclusion criteria were as follows: (1) duplicate articles from the same authors or laboratories or conducted among the same populations; (2) conference reports or reviews; (3) without control group; and (4) data could not be extracted.

### Data extraction

Two of our authors independently assessed the quality of selected studies. Each item of a single study should be achieved a final consensus. The extracted information was as follow: the name of first author, published year, country, ethnicity, mean age, sample size, genotype method, alleles and genotypes distribution, source of cases and controls, and HWE of genotypes in controls.

### Statistical analysis

The pooled odds ratio (OR) with its 95 % confidence interval (CI) were employed to estimate the association between ADH1B Arg47His variant and esophageal carcinoma susceptibility. The *Z* test was used to determine the significance of ORs, and *P* value was less than 0.05 considered as statistically significance. The allelic model (His vs. Arg), the homologous model (His/His vs. Arg/Arg), the heterogeneous model (Arg/His vs. Arg/Arg), the dominant model (His/His+Arg/His vs. Arg/Arg) and the recessive model (His/His vs. Arg/His+Arg/Arg) were examined. The *Q* test and the *I*^2^ test were used to assess the degree and proportion of between-study heterogeneity, respectively. The fixed-effect model was used when the *P* value of the *Q* test was more than 0.10 and the *I*^2^ of the *I*^2^ test was less than 50 %; otherwise, the random-effect model was used. All statistical analyses were conducted in Review Manager 5.2 (the Cochrane Collaboration, Oxford, England). All the tests were two-sided.

## Results

### Characteristics of included studies

**Fig. 1:**
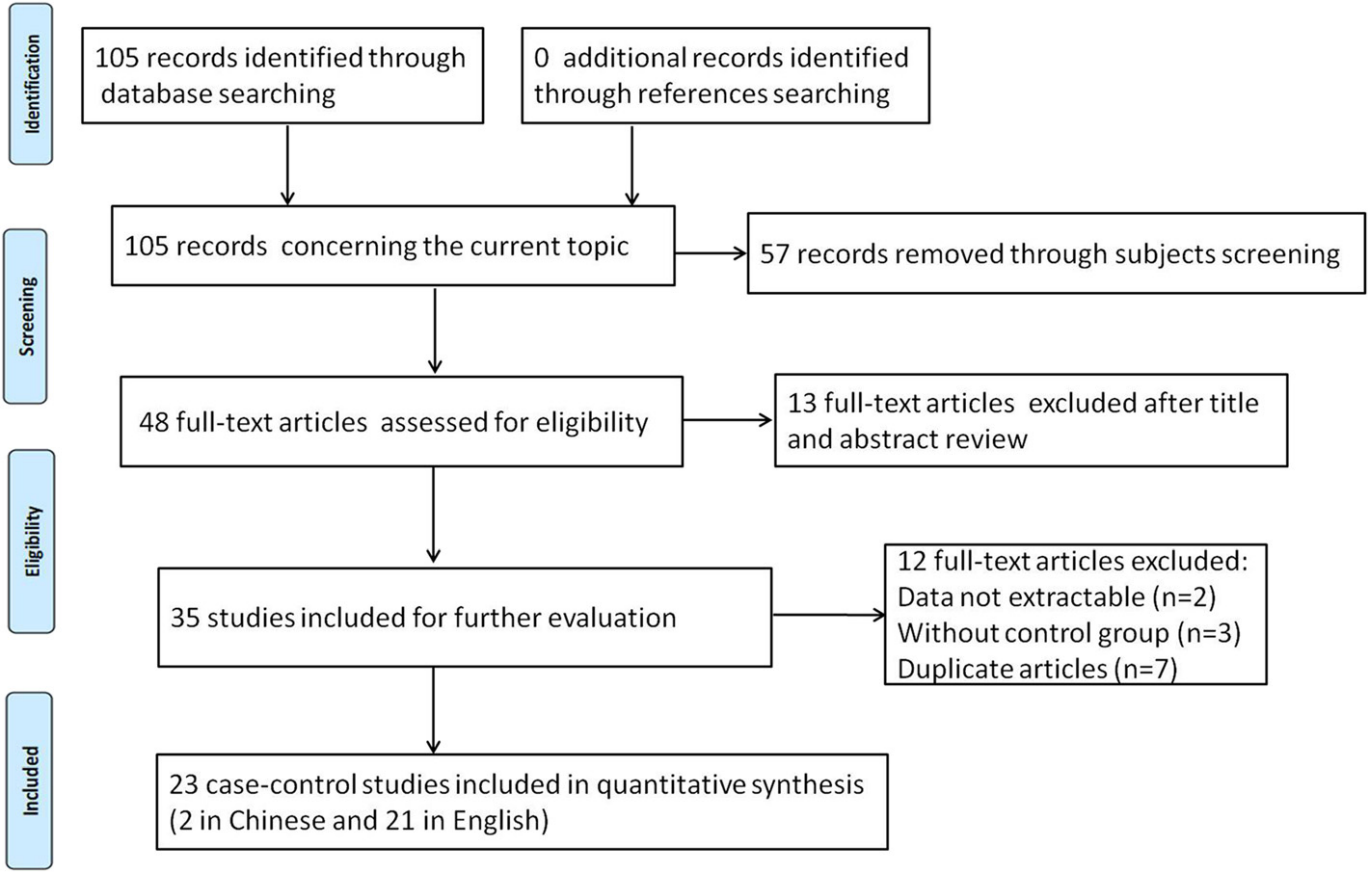
Flow diagram of the selection process

We firstly identified 105 articles. After the inclusion and exclusion criteria filtering, a total of 23 relevant articles were finally screened out, including 9338 esophageal cancer patients and 14,896 matched controls. Figure 1 presented the selection process. Of the 23 articles, two were written in Chinese [33, 34] and 21 were in English [35–55]. The participants of Hashibe et al.’s study were from five countries (Romania, Poland, Russia, Slovakia, and Czech Republic) [38], while other studies were from seven countries (Japan, Thailand, Iran, Kashmir, Sudan, Dutch and China), respectively. There were two sources of controls: population-based controls and hospital-based controls. The ADH1B Arg47His variant was measured in eight methods. Table 1 listed the main characteristics of included studies. Table 2 displayed the alleles and genotypes information of ADH1B Arg47His variant in each included studies.

**Table 1:** Main characteristics of included studies in this meta-analysis

First author	Year	Country	Ethnicity	Mean age		Sample size		Source of		Genotyping methods
				Cases	Controls	Cases	Controls	Cases	Controls	
Yokoyama A	2001	Japan	Asian	56 ± 7	53 ± 8	112	526	ESCC	HB	PCR-RFLP
Boonyaphiphat P	2002	Thailand	Asian	64.0 ± 9.7	64.5 ± 12.3	202	261	ESCC	HB	APLP
Chao YC	2003	China (Taiwan)	Asian	64.6 ± 12.3	53.0 ± 18.8	88	105	EC	HB	PCR-RFLP
Yokoyama T	2003	Japan	Asian	61.7 ± 7.9	58.9 ± 7.1	233	610	ESCC	PB	PCR-RFLP
Chen HG	2005	China	Asian	58.06 (35–80)	58.53 (35–82)	137	137	EC	PB	PCR
Yang CX	2005	Japan	Asian	61.4 ± 0.6	61.4 ± 0.4	165	495	ESCC/EAC	HB	PCR-CTPP
Hashibe M	2006	Mixed	Caucasian	56.7 ± 2.1	58.9 ± 3.4	167	887	ESCC	PB	TaqMan
Yang SJ	2007	China	Asian	58.3 ± 8.3	52.8 ± 13.2	191	198	EC	PB	PCR-CTPP
Guo YM	2008	China	Asian	60.2 ± 8.9	59.7 ± 9.7	80	480	ESCC	PB	PCR-RFLP
Lee CH	2008	China (Taiwan)	Asian	60.2 ± 3.8	61.3 ± 3.9	406	656	ESCC	HB	PCR-RFLP
Akbai	2009	Iran	Asian	63.6 (25–89)	55.2 (24–90)	746	1373	ESCC	PB	Sequenom
Cui R	2009	Japan	Asian	64.2 ± 8.7	59.7 ± 16.2	1070	2836	ESCC	PB	BeadChip
Ding JH	2010	China	Asian	68.2 ± 5.6	69.8 ± 7.3	221	191	EC	PB	PCR-DHPLC
Tanaka F	2010	Japan	Asian	67.2 ± 7.3	66.5 ± 7.2	742	820	OSCC	PB	BRLMM
Iqbal B	2011	Kashmir	Asian	NA	NA	50	50	EC	PB	Sequencing
Wang YL	2011	China	Asian	57.2 ± 9.4	56.1 ± 7.3	81	162	EC	PB	PCR-CTPP
Gu HY	2012	China	Asian	62.5 ± 6.2	63.8 ± 7.1	380	380	ESCC	HB	MALDI-ToF-MS
Dura P	2013	Dutch	Caucasian	65.0 ± 10.9	64.9 ± 11.1	351	430	ESCC/EAC	PB	TaqMan
Gao Y	2013	China	Asian	NA	NA	2139	2273	ESCC	PB	TaqMan
Wu M	2013	China	Asian	63.7 ± 9.4	63.7 ± 10.3	846	1079	EC	PB	Sequencing
Hou AM	2014	China	Asian	56.5 ± 5.5	58.7 ± 6.3	110	110	ESCC/EAC	PB	PCR-RFLP
Ye B	2014	China	Asian	58.7 ± 6.4	59.9 ± 7.2	1001	1391	ESCC	HB	PCR-RFLP
Babiker H	2015	Sudan	Africa	56.41	47.87	134	233	ESCC/EAC	PB	PCR-RFLP

*NA* not available, *ESCC* esophageal squamous cell carcinoma, *EAC* esophageal adenocarcinoma, *OSCC* oesophageal squamous cell carcinoma, *HB* hospital-based, *PB* population-based, *NP* normal healthy population, *APLP* amplified product length polymorphism method, *PCR-CTPP* polymerase chain reaction with the confronting two-pair primer, *PCR-DHPLC* polymerase chain reaction and denaturing high-performance liquid chromatography, *BRLMM* Bayesian robust linear model with Mahalanobis algorithm, *MALDI-ToF-MS* matrix-assisted laser desorption/ionization-time-of-flight mass spectrometry

**Table 2:** Distribution of alleles and genotypes in each included studies

First author	Cases					Controls					
	Arg/Arg	Arg/His	His/His	Arg	His	Arg/Arg	Arg/His	His/His	Arg	His	HWE
	(*1/*1)	(*1/*2),	(*2/*2)	(*1)	(*2)	(*1/*1)	(*1/*),	(*2/*2)	(*1)	(*2)	
Yokoyama A	56	56*		–	–	145	381*			–	–
Boonyaphiphat P	101	86	15	288	116	94	139	28	327	195	0.08
Chao YC	19	41	28	79	97	7	43	55	57	153	0.94
Yokoyama T	51	73	109	175	291	31	212	367	274	946	0.99
Chen HG	26	54	57	106	168	14	49	74	77	197	0.40
Yang CX	6	85	74	97	233	22	168	304	212	776	0.98
Hashibe M	163	4*		–	–	792	95*		–	–	–
Yang SJ	33	80	78	146	236	22	76	100	120	276	0.44
Guo YM	17	25	38	59	101	24	168	288	216	744	0.99
Lee CH	117	149	140	383	429	46	275	335	367	945	0.59
Akbai	21	232	490	274	1212	73	471	827	617	2125	0.86
Cui R	194	363	510	751	1383	151	986	1626	1288	4238	0.99
Ding JH	19	96	106	134	308	8	75	108	91	291	0.53
Tanaka F	149	237	356	535	949	49	287	484	385	1255	0.76
Iqbal B	12	36	2	60	40	12	37	1	61	39	0.30
Wang YL	15	34	33	64	100	17	67	78	101	223	0.90
Gu HY	53	168	158	274	484	26	170	182	222	534	0.26
Dura P	326	20	0	672	20	406	23	0	835	23	0.85
Gao Y	252	907	939	1411	2785	199	909	1155	1307	3219	0.57
Wu M	138	309	355	585	1019	101	410	510	612	1430	0.38
Hou AM	59*		51	–	–	48*		62	–	–	–
Ye B	224	400	377	848	1154	150	578	663	878	1904	0.36
Babiker H	115	8	2	238	12	188	18	4	394	26	0.06

*HWE* Hardy-Weinberg Equilibrium;–, not applicable; *for Yokoyama A and Hashibe M means His/His+Arg/His; *for Hou AM means Arg/His+Arg/Arg

### Overall association between ADH1B Arg47His polymorphism and esophageal cancer risk

**Fig. 2:**
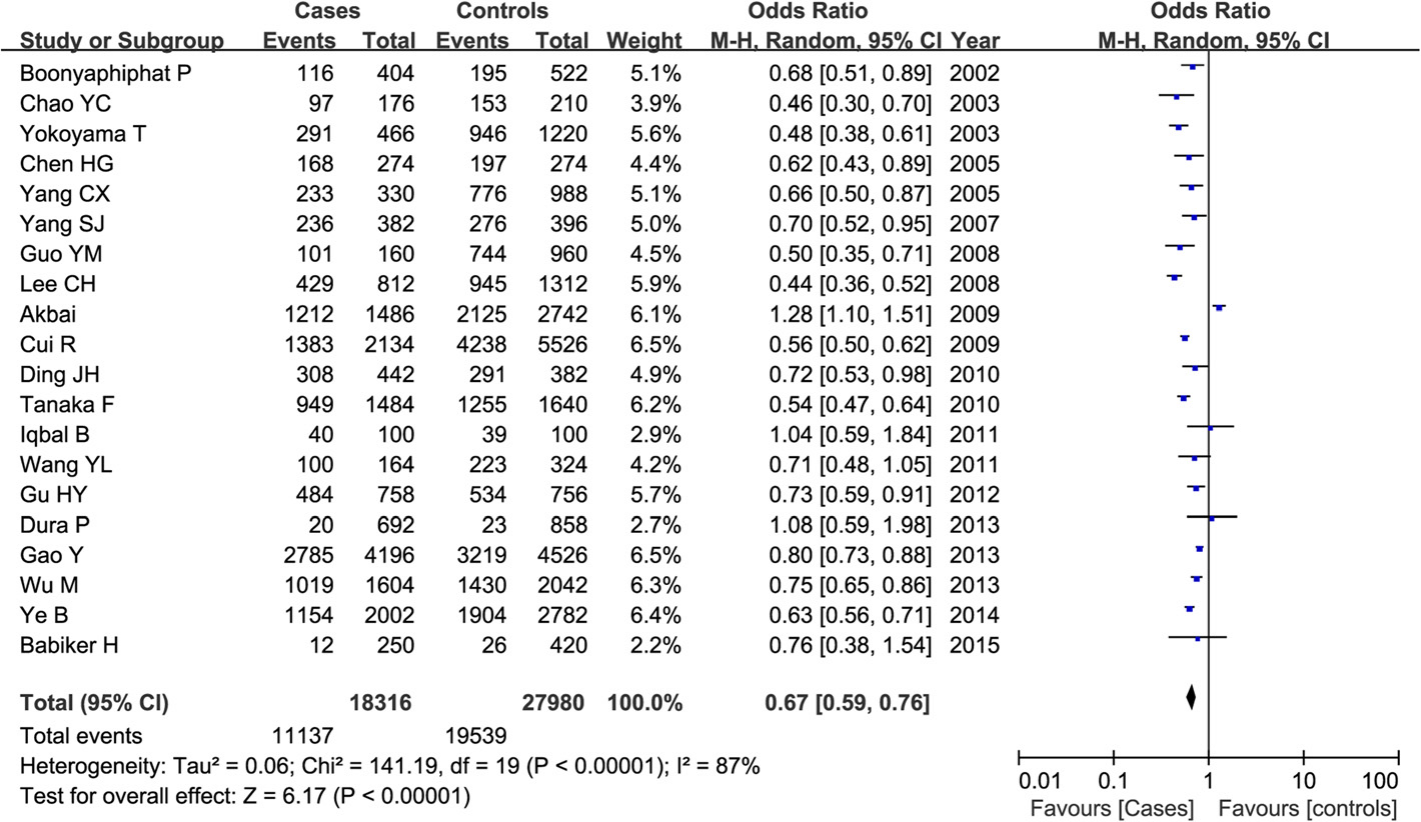
Meta-analysis of ADH1B Arg47His polymorphism and esophageal cancer risk in a random-effect model under the allelic models (His vs. Arg)

**Fig. 3:**
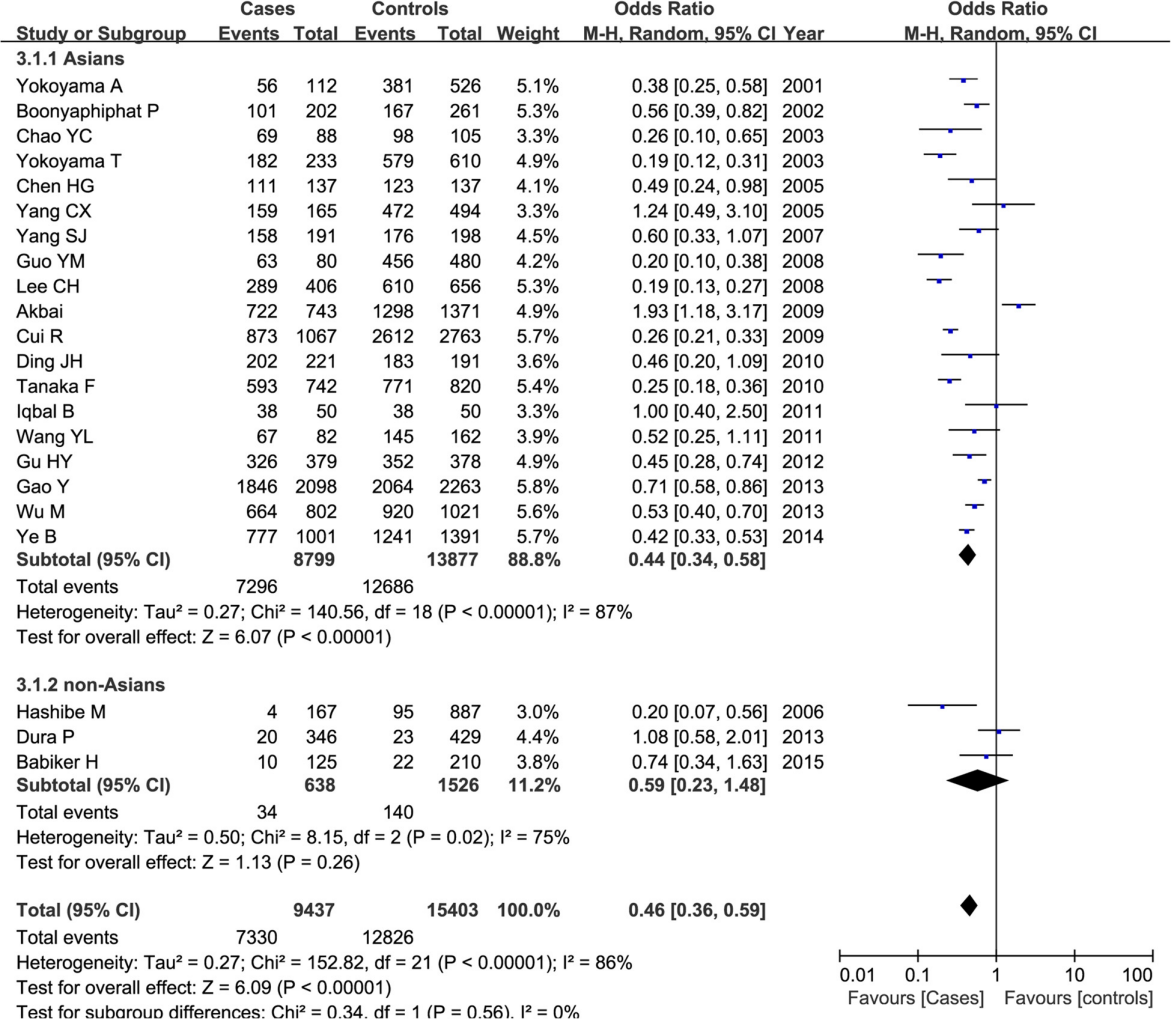
Subgroup analysis by ethnicity of ADH1B Arg47His polymorphism and esophageal cancer susceptibility under the dominant model (His/His+Arg/His vs. Arg/Arg)

**Fig. 4:**
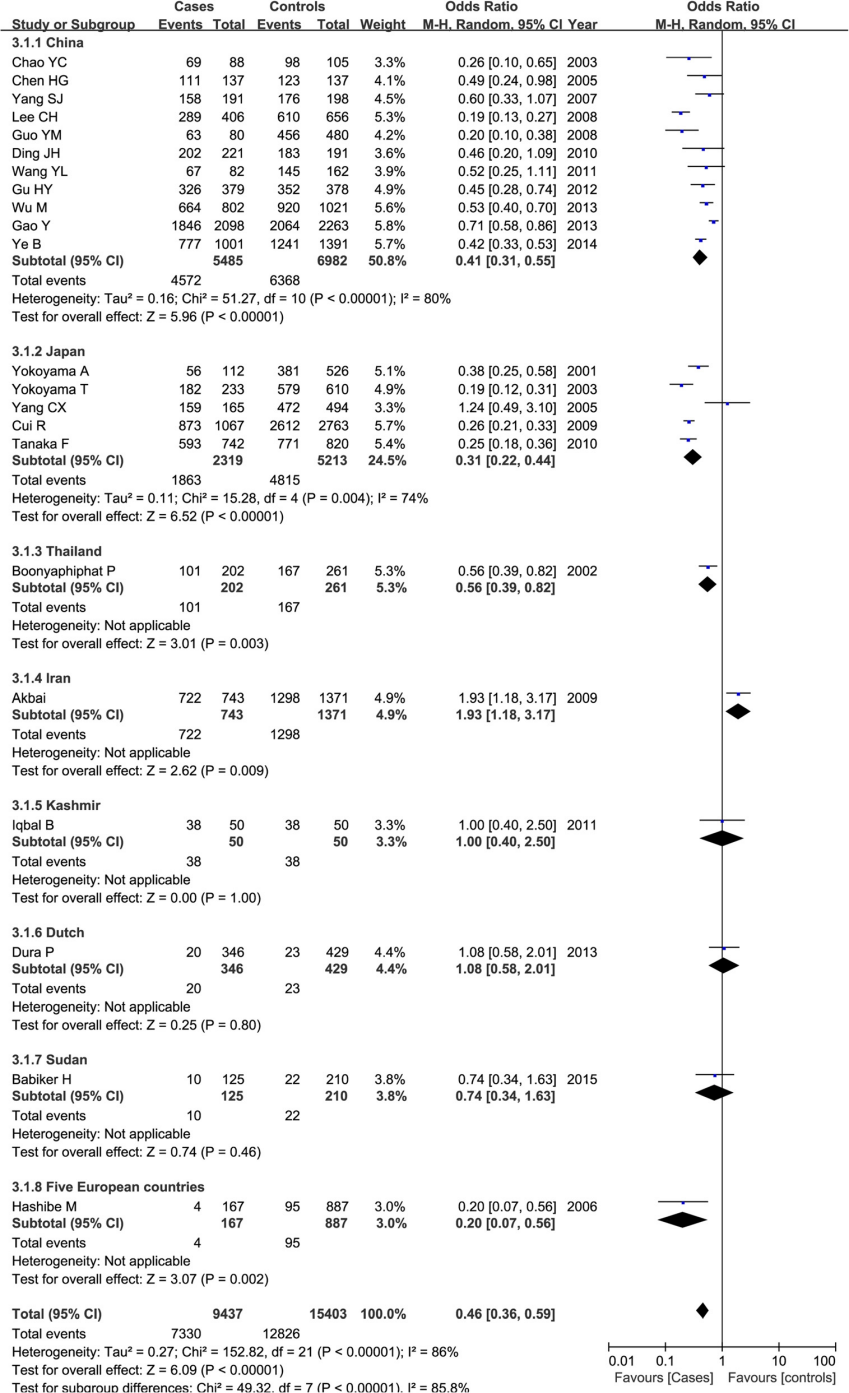
Association between ADH1B Arg47His polymorphism and esophageal cancer under the dominant model based on countries

A significant heterogeneity among all the included studies was observed (*P* < 0.01 and *I*^2^ > 50 %), and the random-effect model was used. Table 3 listed the results of the association between the ADH1B Arg47His polymorphism and esophageal cancer susceptibility. Overall, we found that the frequency of 47His allele was higher than the 47Arg allele in both cases and controls, and the statistical analysis demonstrated that the 47His allele was significant associated with the decreased risk of esophageal cancer in total populations (His vs. Arg: OR = 0.67, 95 % CI = 0.59–0.76, *P* < 0.00001) as shown in Fig. 2. This significant protective relationship was also identified under other genetic models (*P* < 0.00001). Subgroup analysis by ethnicity showed that this polymorphism revealed an ethnic difference and geographic variance. In Asians, ADH1B 47His variant was shown to be associated with the decreased esophageal cancer risk under all the genetic models (*P* < 0.00001); while in non-Asians, no significant correlation was detected in any genetic models (*P* > 0.05). Figure 3 showed the result of the His carriers of the His/His and Arg/His genotypes compared with the Arg/Arg genotype in both groups. Furthermore, we also considered the role of this genetic variant in esophageal cancer among different countries. There were 12 articles in China (5485 patients and 6982 controls), five in Japan (2319 patients and 5213 controls), and six in other countries. Our result demonstrated that ADH1B Arg47His variant was a protective factor for cancer risk in Chinese and Japanese populations as shown in Fig. 4.

**Table 3:** Meta-analysis of the overall association between ADH1B Arg47His polymorphism and esophageal cancer risk

Classification	Comparisons	Number of included studies	Test of association		Test of heterogeneity		
			OR (95 % CI)	*P*	Ph	I^2^	Model
Total	His vs. Arg	20	0.67 (0.59, 0.76)	<0.00001	<0.00001	87 %	R
	His/His vs. Arg/Arg	20	0.40 (0.30, 0.54)	<0.00001	<0.00001	86 %	R
	Arg/His vs. Arg/Arg	20	0.52 (0.40, 0.68)	<0.00001	<0.00001	85 %	R
	His/His+Arg/His vs. Arg/Arg	22	0.46 (0.36, 0.59)	<0.00001	<0.00001	86 %	R
	His/His vs. Arg/His+Arg/Arg	21	0.69 (0.61, 0.77)	<0.00001	<0.00001	69 %	R
Asian	His vs. Arg	18	0.66 (0.57, 0.75)	<0.00001	<0.00001	88 %	R
	His/His vs. Arg/Arg	18	0.40 (0.30, 0.54)	<0.00001	<0.00001	87 %	R
	Arg/His vs. Arg/Arg	18	0.49 (0.38, 0.65)	<0.00001	<0.00001	86 %	R
	His/His+Arg/His vs. Arg/Arg	19	0.44 (0.34, 0.58)	<0.00001	<0.00001	87 %	R
	His/His vs. Arg/His+Arg/Arg	19	0.68 (0.61, 0.77)	<0.00001	<0.00001	71 %	R
Non-Asian	His vs. Arg	2	0.93 (0.59, 1.47)	0.75	0.46	0 %	F
	His/His vs. Arg/Arg	1	0.82 (0.15, 4.53)	0.82	NA	NA	
	Arg/His vs. Arg/Arg	2	0.95 (0.57, 1.56)	0.83	0.46	0 %	F
	His/His+Arg/His vs. Arg/Arg	3	0.59 (0.23, 1.48)	0.26	0.02	75 %	R
	His/His vs. Arg/His+Arg/Arg	1	0.84 (0.15, 4.64)	0.84	NA	NA	
China	His vs. Arg	11	0.64 (0.56, 0.73)	<0.00001	<0.00001	78 %	R
	His/His vs. Arg/Arg	11	0.37 (0.29, 0.50)	<0.00001	<0.00001	79 %	R
	Arg/His vs. Arg/Arg	11	0.47 (0.35, 0.63)	<0.00001	<0.00001	77 %	R
	His/His+Arg/His vs. Arg/Arg	11	0.41 (0.31, 0.55)	<0.00001	<0.00001	80 %	R
	His/His vs. Arg/His+Arg/Arg	12	0.71 (0.66, 0.76)	<0.00001	0.16	29 %	F
Japan	His vs. Arg	4	0.55 (0.51, 0.60)	<0.00001	0.41	0 %	F
	His/His vs. Arg/Arg	4	0.26 (0.18, 0.38)	<0.00001	0.03	66 %	R
	Arg/His vs. Arg/Arg	4	0.35 (0.21, 0.59)	<0.00001	0.0008	82 %	R
	His/His+Arg/His vs. Arg/Arg	5	0.31 (0.22, 0.44)	<0.00001	0.004	74 %	R
	His/His vs. Arg/His+Arg/Arg	4	0.62 (0.56, 0.69)	<0.00001	0.65	0 %	F

*NA* not applicable, *OR* odds ratio, *95 % CI* 95 % confidence interval, *Ph and I* ^*2*^ test of heterogeneity, *F* fixed-effect model, *R* random-effect model

### Combined effect of ADH1B Arg47His variant and alcohol drinking, tobacco smoking, and gender difference on esophageal cancer risk

**Fig. 5:**
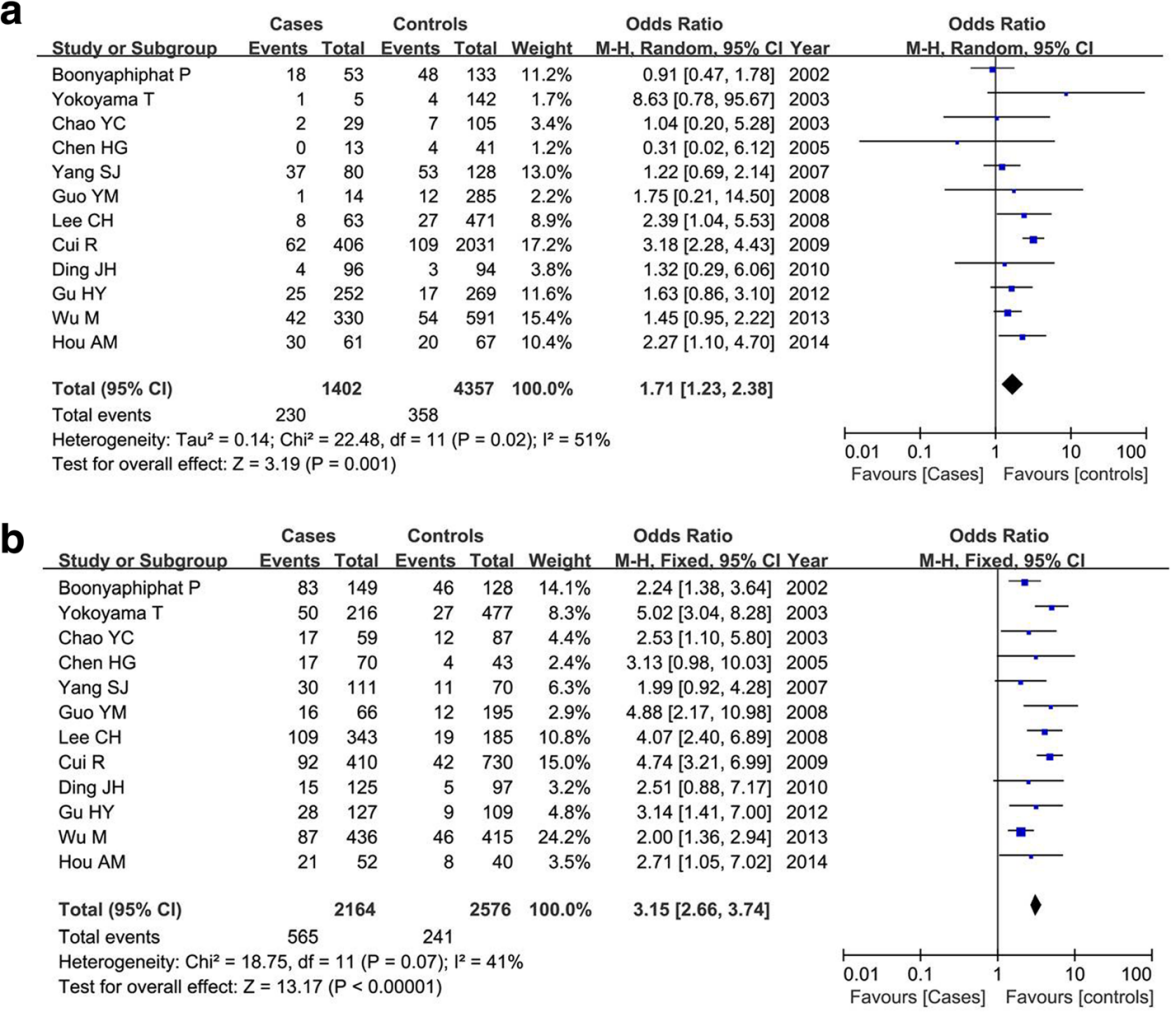
Forest plot of the combined effect of ADH1B Arg47His polymorphism and alcohol drinking condition and esophageal cancer risk under the Arg/Arg versus Arg/His+His/His model (**a** non-drinkers; **b** drinkers)

Fifteen studies estimated the combined effect of ADH1B Arg47His polymorphism and alcohol drinking on esophageal cancer risk. However, the relevant data could be extracted only from 12 studies, including 3545 patients and 6909 controls. Based on these data, we could only assess the combined effect on esophageal cancer susceptibility under the Arg/Arg versus His/His+Arg/His model. We divided the subjects into two groups: non-drinking group (1134 cases and 3526 controls) and drinking group (2411 cases and 3383 controls). Our results found that Arg/Arg genotype compared with the His carrier of His/His and Arg/His genotypes was significantly associated with the increased higher risk of esophageal cancer in the drinking group (OR = 3.15, 95 % CI = 2.66–3.74, *P* < 0.00001) and lower in the non-drinking group (OR = 1.71, 95 % CI = 1.23–2.38, *P* = 0.001) as shown in Fig. 5.

**Fig. 6:**
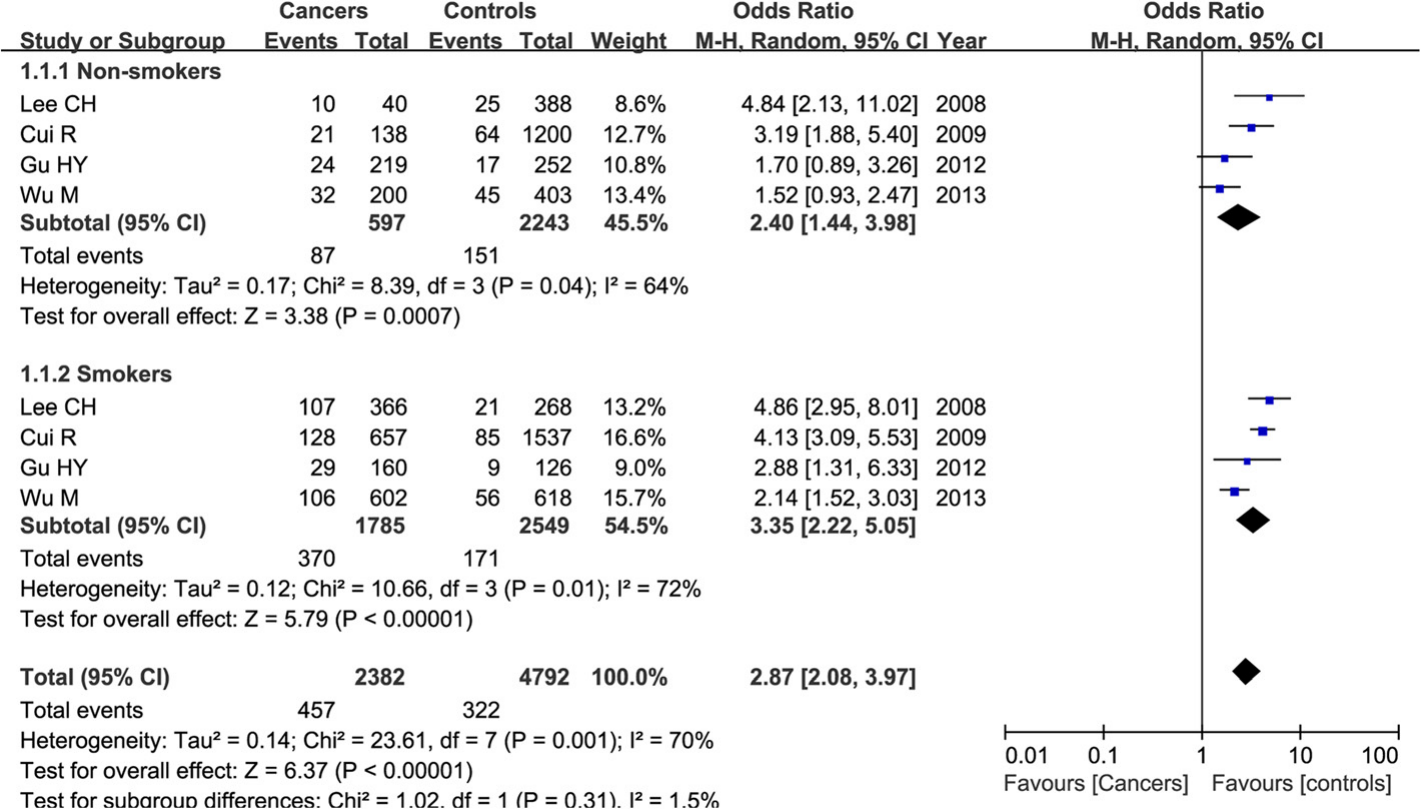
Meta-analysis of the combined effect of ADH1B Arg47His polymorphism and smoking status and esophageal cancer risk under the Arg/Arg versus Arg/His+His/His model

Four articles considered the combined effect of ADH1B Arg47His polymorphism and tobacco smoking on esophageal cancer susceptibility, containing 2382 patients and 4792 controls. Our result demonstrated that the Arg/Arg genotype was associated with esophageal cancer occurrence in both non-smokers (OR = 2.40, 95 % CI = 1.44–3.98, *P* = 0.0007) and smokers (OR = 3.35, 95 % CI = 2.22–5.05, *P* < 0.00001) in the random-effect model as shown in Fig. 6, and this relationship was a bit stronger in smokers than that in non-smokers.

**Fig. 7:**
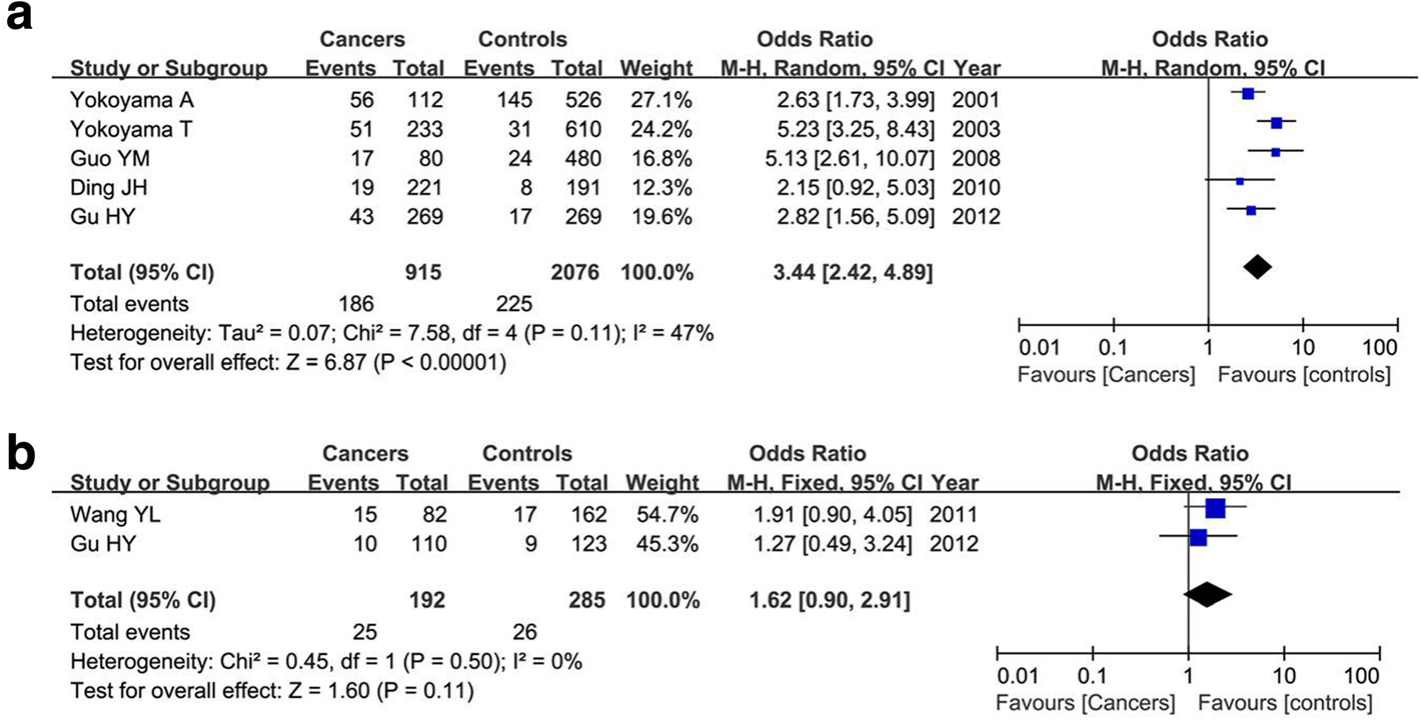
Forest plot of the combined effect of ADH1B Arg47His polymorphism and gender variance and esophageal cancer risk under the Arg/Arg versus Arg/His+His/His model (**a** males; **b** females)

Six articles were selected for gender variance (five for males and two for females). Our result showed that the Arg/Arg genotype of ADH1B Arg47His variant increased the esophageal cancer risk in male patients (OR = 3.44, 95 % CI = 2.42–4.89, *P* < 0.0001), while not in female patients (OR = 1.62, 95 % CI = 0.90–2.91, *P* = 0.11) as shown in Fig. 7.

### Sensitivity analysis and publication bias

**Fig. 8:**
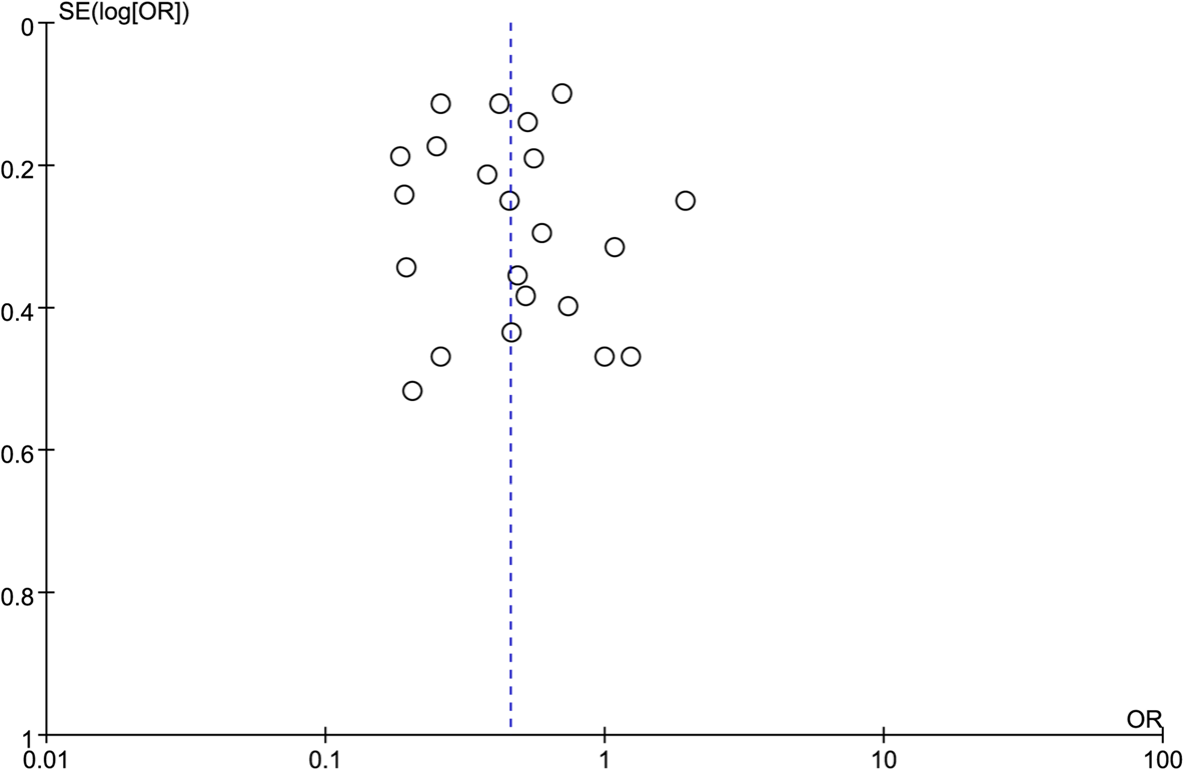
Funnel plot of the publication bias in this meta-analysis under the dominant model in total population (His/His+Arg/His vs. Arg/Arg)

We deleted a single included study at a time to verify whether our results were influenced by each study. The result showed that even though the between-study heterogeneity was reduced, the pooled ORs were not significantly changed, indicating that there was no publication bias in the present meta-analysis. The funnel plot among the total population under the dominant model further reveal no publication bias as shown in Fig. 8.

## Discussion

In this meta-analysis, we totally screened out 23 relevant articles. Our results showed that the ADH1B Arg47His variant was correlated with the decreased occurrence of esophageal cancer under all the genetic models among total population. Subgroup analysis by ethnicity demonstrated that this variant was associated with the decreased cancer risk in Asian populations as well, especially in Chinese and Japanese. However, in non-Asian population, no significant relationship was observed. Furthermore, we found that the combined effect of Arg/Arg genotype in ADH1B Arg47His variant and alcohol drinking, tobacco smoking, and male patients revealed a higher risk on esophageal cancer risk when compared with the His carrier of His/His and Arg/His genotypes, respectively. Our results were consistent with previous meta-analyses which showed that ADH1B*47Arg could significantly increase the risk of ESCC in Asians, especially when coupled with alcohol drinking [29–31].

Esophageal cancer is considered as a serious malignancy worldwide for its extremely aggressive nature and poor survival rate [56]. It is a multistep, multifactorial disease, and the incidence is varied due to the geographical diversities; thus, the therapeutic management might be different as well. Genetic variations might be involved in esophageal cancer development and predict the prognosis [57]. Studies have shown that ADH gene might play a role in carcinogenesis. ADH constitutes a large family of enzymes and is a metabolic barrier against self-administer ethanol [58]. ADH isoenzyme is responsible for the reversible oxidation of alcohols to acetaldehyde, which is in turn oxidized to aldehyde by aldehyde dehydrogenases (ALD) [59]. ADH1B and ALDH2 are the major enzymes involved in the alcohol-metabolizing pathways in humans. ADH1B is one member of ADH family and is expressed in the liver and presented in gastrointestinal tract. This gene is mainly contributed for ethanol to carcinogenic metabolite conversion during the elimination phage [60]. In addition, ADH1B expression could result in high blood acetaldehyde levels, which can easily give rise to DNA damage, and finally cause the cancer occurrence [61]. Genetic polymorphisms of ADH1B could modulate exposure levels to ethanol. ADH1B can include three alleles (ADH2*1, ADH2*2, and ADH2*3). Among which, the Arg47His variant (ADH2*2) was the most studied. The activity of ADH1B was increased by 40-fold in ADH1B His/His individuals [62], resulting in the fast accumulation of acetaldehyde.

SNPs of ADH1B gene (the major alcohol-metabolizing enzyme gene) may modify the effects of alcohol on metabolic and clinical phenotypes [63]. Blood ethanol concentrations of ADH1B*2/*2 group were shown higher than those of ADH1B*1/*2 group [64]. ADH1B allele was associated with a reduction in alcohol consumption [65] and might be an important protective factor for alcoholic liver cirrhosis, especially for Asians [66]. ADH2*2 predicted less drinking and was a protective effect against heavy drinking in Jewish samples [67]. Heavy alcohol consumption and heavy smoking were shown to be associated with the increased risk of esophageal cancer particularly in individuals with the flushing response [68].

Previous studies have found that ADH1B Arg47His polymorphism was associated with the pathogenesis of cancers such as colorectal cancer [69] and head and neck cancer [70], while no positive correlation was found in hepatocellular carcinoma [71] and gastric cancer [72]. Several studies have identified the association between ADH1B polymorphism and esophagus cancer susceptibility. Chen et al. have observed that individuals with ADH1BArg/Arg genotype had a 3.99-fold risk of developing esophageal cancer compared with those with ADH1B His/His genotype [73]. Li et al. have demonstrated that ADH2*1 was associated with the increased risk of oesophageal cancer, possibly due to the tolerance of the carriers of these alleles to alcohol consumption compared to those with high-activity alleles ADH2*2 which are associated with higher production of the unpleasant acetaldehyde intermediate [74]. Oze et al. found that the magnitude of effect of ADH1B polymorphisms was greater in subjects who were heavy drinkers, heavy smokers, and had esophageal cancer [75]. Consumption of tobacco and alcohol, coupled with ADH1B genotypes, determines a substantial magnitude of tumorigenetic effect on earlier age ESCC diagnosis [76].

Several limitations were presented in this meta-analysis. Firstly, most of the included studies were conducted in the Asian population, while other ethnicities should be considered in the future. Secondly, the stages of patients with esophageal cancer could not be extracted from the included studies, which might limit the application of our results. Thirdly, the combined effect of ADH1B Arg47His variant and age group also should be focused on. Lastly, other polymorphisms in ADH1B gene or other ADH genes which might alter the metabolism of alcohol should be included.

## Conclusions

In conclusion, our results suggested that ADH1B Arg47His polymorphism might be a protective factor on esophageal cancer susceptibility in Asians. The Arg/Arg genotypes combined with alcohol drinking, tobacco smoking, and males might be strongly increased the risk of esophageal cancer. However, further studies with more ethnicities should be taken into account in future researches.

## Abbreviations

AC, adenocarcinomas; ADH, alcohol dehydrogenase gene; ADH1B, alcohol dehydrogenase 1B; ALD, aldehyde dehydrogenases; CI, confidence interval; OR, odds ratio; SCC, squamous cell carcinomas; SNPs, single nucleotide polymorphisms

## References

[1] Schweigert M, Dubecz A, Stein HJ (2013). Oesophageal cancer—an overview. Nat Rev Gastroenterol Hepatol.

[2] Napier KJ, Scheerer M, Misra S (2014). Esophageal cancer: a review of epidemiology, pathogenesis, staging workup and treatment modalities. World J Gastrointest Oncol.

[3] Stewart BW, Wild CP. World cancer report 2014. Geneva: International Agency for Research on Cancer; 2014.

[4] Kornblum N. Esophageal Cancer[M], Geriatric Gastroenterology. New York: Springer; 2012. p. 571-579.

[5] Arnal MJD, Arenas ÁF, Arbeloa ÁL (2015). Esophageal cancer: risk factors, screening and endoscopic treatment in Western and Eastern countries. World J Gastroenterol.

[6] Gao Y-B, Chen Z-L, Li J-G, Hu X-D, Shi X-J, Sun Z-M, Zhang F, Zhao Z-R, Li Z-T, Liu Z-Y. Genetic landscape of esophageal squamous cell carcinoma. Nat Genet. 2014. 10.1038/ng.3076 25151357

[7] Tang W, Chen Z, Lin K, Su M, Au W (2015). Development of esophageal cancer in Chaoshan region, China: association with environmental, genetic and cultural factors. Int J Hyg Environ Health.

[8] Whiteman DC, Sadeghi S, Pandeya N, Smithers BM, Gotley DC, Bain CJ, Webb PM, Green AC (2008). Combined effects of obesity, acid reflux and smoking on the risk of adenocarcinomas of the oesophagus. Gut.

[9] Ferlay J, Soerjomataram I, Dikshit R, Eser S, Mathers C, Rebelo M, Parkin DM, Forman D, Bray F (2015). Cancer incidence and mortality worldwide: sources, methods and major patterns in GLOBOCAN 2012. Int J Cancer.

[10] Giri S, Pathak R, Aryal MR, Karmacharya P, Bhatt VR, Martin MG (2015). Incidence trend of esophageal squamous cell carcinoma: an analysis of Surveillance Epidemiology, and End Results (SEER) database. Cancer Causes Control.

[11] Andrici J, Eslick GD. Epidemiology and Risk Factors for Esophageal Cancer[M], Esophageal Cancer. Heidelberg: Springer International Publishing; 2015. p. 1-23.

[12] Siegel RL, Miller KD, Jemal A (2015). Cancer statistics, 2015. CA Cancer J Clin.

[13] Merkow RP, Bilimoria KY, Keswani RN, Chung J, Sherman KL, Knab LM, Posner MC, Bentrem DJ (2014). Treatment trends, risk of lymph node metastasis, and outcomes for localized esophageal cancer. J Natl Cancer Inst.

[14] Lao-Sirieix P, Fitzgerald RC (2012). Screening for oesophageal cancer. Nat Rev Clin Oncol.

[15] Kaz AM, Grady WM (2014). Epigenetic biomarkers in esophageal cancer. Cancer Lett.

[16] Pennathur A, Gibson MK, Jobe BA, Luketich JD (2013). Oesophageal carcinoma. Lancet.

[17] Whiteman DC (2014). Esophageal cancer: priorities for prevention. Curr Epidemiol Rep.

[18] Chattopadhyay I (2014). A brief overview of genetics of esophageal squamous cell carcinoma. J Cell Sci Molecul Biol.

[19] Zuo L, Wang K, Zhang X-Y, Pan X, Wang G, Tan Y, Zhong C, Krystal JH, Zhang H, Luo X (2013). Association between common alcohol dehydrogenase gene (ADH) variants and schizophrenia and autism. Hum Genet.

[20] Luo X, Kranzler HR, Zuo L, Zhang H, Wang S, Gelernter J (2008). ADH7 variation modulates extraversion and conscientiousness in substance-dependent subjects. Am J Med Genet B Neuropsychiatr Genet.

[21] Svensson S, Some M, Lundsjö A, Helander A, Cronholm T, Höög JO (1999). Activities of human alcohol dehydrogenases in the metabolic pathways of ethanol and serotonin. Eur J Biochem.

[22] Strommer J (2011). The plant ADH gene family. Plant J.

[23] Yoshida A (1991). Genetics of human alcohol-metabolizing enzymes. Prog Nucleic Acid Res Mol Biol.

[24] Bosron WF, Li TK (1986). Genetic polymorphism of human liver alcohol and aldehyde dehydrogenases, and their relationship to alcohol metabolism and alcoholism. Hepatology.

[25] Bierut LJ, Goate AM, Breslau N, Johnson EO, Bertelsen S, Fox L, Agrawal A, Bucholz KK, Grucza R, Hesselbrock V (2012). ADH1B is associated with alcohol dependence and alcohol consumption in populations of European and African ancestry. Mol Psychiatry.

[26] Li D, Zhao H, Gelernter J (2011). Strong association of the alcohol dehydrogenase 1B gene (ADH1B) with alcohol dependence and alcohol-induced medical diseases. Biol Psychiatry.

[27] Ito H, Oze I, Hosono S, Watanabe M, Tanaka H, Matsuo K (2015). The risk prediction for esophageal cancer by drinking, smoking, and the polymorphisms of ALDH2 and ADH1B. Cancer Res.

[28] Ma W-J, Lv G-D, Zheng S-T, Huang C-G, Liu Q, Wang X, Lin R-Y, Sheyhidin I, Lu X-M (2010). DNA polymorphism and risk of esophageal squamous cell carcinoma in a population of North Xinjiang, China. World J Gastroenterol.

[29] Zhang L, Jiang Y, Wu Q, Li Q, Chen D, Xu L, Zhang C, Zhang M, Ye L (2014). Gene–environment interactions on the risk of esophageal cancer among Asian populations with the G48A polymorphism in the alcohol dehydrogenase-2 gene: a meta-analysis. Tumor Biol.

[30] Zhang G, Mai R, Huang B. ADH1B Arg47His polymorphism is associated with esophageal cancer risk in high-incidence Asian population: evidence from a meta-analysis. PLoS One. 2010;5. 10.1371/journal.pone.0013679 PMC2965113 21048924

[31] Yang S-J, Yokoyama A, Yokoyama T, Huang Y-C, Wu S-Y, Shao Y, Niu J, Wang J, Liu Y, Zhou X-Q (2010). Relationship between genetic polymorphisms of ALDH2 and ADH1B and esophageal cancer risk: a meta-analysis. World J Gastroenterol.

[32] Zhang H-Z, Jin G-F, Shen H-B (2012). Epidemiologic differences in esophageal cancer between Asian and Western populations. Chin J Cancer.

[33] Hou A, Tong X, Miao Z. Correlation of alcohol dehydrogenase and aldehyde dehydrogenase polymorphisms, and alcohol drinking in esophageal cancer. China J Pharm Econ. 2014;223–224.

[34] Chen H. A match case–control study on esophageal cancer susceptibility with lifestyle habits and genetic polymorphisms of ALDH2 and ADH2. Sichuan University; Master’s degree; 2005.

[35] Cui R, Kamatani Y, Takahashi A, Usami M, Hosono N, Kawaguchi T, Tsunoda T, Kamatani N, Kubo M, Nakamura Y (2009). Functional variants in ADH1B and ALDH2 coupled with alcohol and smoking synergistically enhance esophageal cancer risk. Gastroenterology.

[36] Lee CH, Lee JM, Wu DC, Goan YG, Chou SH, Wu I, Kao EL, Chan TF, Huang MC, Chen PS (2008). Carcinogenetic impact of ADH1B and ALDH2 genes on squamous cell carcinoma risk of the esophagus with regard to the consumption of alcohol, tobacco and betel quid. Int J Cancer.

[37] Tanaka F, Yamamoto K, Suzuki S, Inoue H, Tsurumaru M, Kajiyama Y, Kato H, Igaki H, Furuta K, Fujita H (2010). Strong interaction between the effects of alcohol consumption and smoking on oesophageal squamous cell carcinoma among individuals with ADH1B and/or ALDH2 risk alleles. Gut.

[38] Hashibe M, Boffetta P, Zaridze D, Shangina O, Szeszenia-Dabrowska N, Mates D, Janout V, Fabiánová E, Bencko V, Moullan N (2006). Evidence for an important role of alcohol-and aldehyde-metabolizing genes in cancers of the upper aerodigestive tract. Cancer Epidemiol Biomarkers Prev.

[39] Wu M, Chang SC, Kampman E, Yang J, Wang XS, Gu XP, Han RQ, Liu AM, Wallar G, Zhou JY (2013). Single nucleotide polymorphisms of ADH1B, ADH1C and ALDH2 genes and esophageal cancer: A population-based case–control study in China. Int J Cancer.

[40] Wang Y, Ji R, Wei X, Gu L, Chen L, Rong Y, Wang R, Zhang Z, Liu B, Xia S (2011). Esophageal squamous cell carcinoma and ALDH2 and ADH1B polymorphisms in Chinese females. Asian Pac J Cancer Prev.

[41] Guo Y-M, Wang Q, Liu Y-Z, Chen H-M, Qi Z, Guo Q-H (2008). Genetic polymorphisms in cytochrome P4502E1, alcohol and aldehyde dehydrogenases and the risk of esophageal squamous cell carcinoma in Gansu Chinese males. World J Gastroenterol.

[42] Gu H, Gong D, Ding G, Zhang W, Liu C, Jiang P, Chen S, Chen Y (2012). A variant allele of ADH1B and ALDH2, is associated with the risk of esophageal cancer. Exp Ther Med.

[43] Akbari MR, Malekzadeh R, Shakeri R, Nasrollahzadeh D, Foumani M, Sun Y, Pourshams A, Sadjadi A, Jafari E, Sotoudeh M (2009). Candidate gene association study of esophageal squamous cell carcinoma in a high-risk region in Iran. Cancer Res.

[44] Babiker H (2015). Genetic polymorphisms in alcohol and xenobiotic metabolizing enzymes as risk factors of oesophageal cancer in Sudan.

[45] Ye B, Ji C-Y, Zhao Y, Li W, Feng J, Zhang X (2014). Single nucleotide polymorphism at alcohol dehydrogenase-1B is associated with risk of esophageal squamous cell carcinoma. Cancer Cell Int.

[46] Gao Y, He Y, Xu J, Xu L, Du J, Zhu C, Gu H, Ma H, Hu Z, Jin G (2013). Genetic variants at 4q21, 4q23 and 12q24 are associated with esophageal squamous cell carcinoma risk in a Chinese population. Hum Genet.

[47] Dura P, Berkers T, van Veen EM, Salomon J, te Morsche RH, Roelofs HM, Kristinsson JO, Wobbes T, Witteman BJ, Tan AC (2013). Polymorphisms in alcohol-metabolizing enzymes and esophageal carcinoma susceptibility: a Dutch Caucasian case–control study. J Hum Genet.

[48] Iqbal B, Dar NAG (2011). Comparison of alcohol dehydrogenase 2 and aldehyde dehydrogenase 2 polymorphism in esophageal cancer cases vs controls in Kashmir.

[49] J-h D, S-p L, H-x C, Wu J-z, Gao C-m, Y-t L, J-n Z, Chang J, Yao G-h (2010). Alcohol dehydrogenase-2 and aldehyde dehydrogenase-2 genotypes, alcohol drinking and the risk for esophageal cancer in a Chinese population. J Hum Genet.

[50] Yang S-J, Wang H-Y, Li X-Q, Du H-Z, Zheng C-J, Chen H-G, Mu X-Y, Yang C-X (2007). Genetic polymorphisms of ADH2 and ALDH2 association with esophageal cancer risk in southwest China. World J Gastroenterol.

[51] Yang C, Matsuo K, Ito H, Hirose K, Wakai K, Saito T, Shinoda M, Hatooka S, Mizutani K, Tajima K (2005). Esophageal cancer risk by ALDH2 and ADH2 polymorphisms and alcohol consumption: exploration of gene-environment and gene-gene interactions. Asian Pac J Cancer Prev.

[52] Chao Y-C, Wang L-S, Hsieh T-Y, Chu C-W, Chang F-Y, Chu H-C (2000). Chinese alcoholic patients with esophageal cancer are genetically different from alcoholics with acute pancreatitis and liver cirrhosis. Am J Gastroenterol.

[53] Boonyaphiphat P, Thongsuksai P, Sriplung H, Puttawibul P (2002). Lifestyle habits and genetic susceptibility and the risk of esophageal cancer in the Thai population. Cancer Lett.

[54] Yokoyama T, Yokoyama A, Kato H, Tsujinaka T, Muto M, Omori T, Haneda T, Kumagai Y, Igaki H, Yokoyama M (2003). Alcohol flushing, alcohol and aldehyde dehydrogenase genotypes, and risk for esophageal squamous cell carcinoma in Japanese men. Cancer Epidemiol Biomarkers Prev.

[55] Yokoyama A, Muramatsu T, Omori T, Yokoyama T, Matsushita S, Higuchi S, Maruyama K, Ishii H (2001). Alcohol and aldehyde dehydrogenase gene polymorphisms and oropharyngolaryngeal, esophageal and stomach cancers in Japanese alcoholics. Carcinogenesis.

[56] Zhang Y (2013). Epidemiology of esophageal cancer. World J Gastroenterol.

[57] Cheung WY, Liu G (2009). Genetic variations in esophageal cancer risk and prognosis. Gastroenterol Clin North Am.

[58] Jelski W, Orywal K, Laniewska M, Szmitkowski M (2010). The diagnostic value of alcohol dehydrogenase (ADH) isoenzymes and aldehyde dehydrogenase (ALDH) measurement in the sera of gastric cancer patients. Clin Exp Med.

[59] De Smidt O, Du Preez JC, Albertyn J (2008). The alcohol dehydrogenases of Saccharomyces cerevisiae: a comprehensive review. FEMS Yeast Res.

[60] Lao-Sirieix P, Caldas C, Fitzgerald RC (2010). Genetic predisposition to gastro-oesophageal cancer. Curr Opin Genet Dev.

[61] Singh NP, Khan A (1995). Acetaldehyde: genotoxicity and cytotoxicity in human lymphocytes. Mut Res DNA Repair.

[62] Lee S-L, Höög J-O, Yin S-J (2004). Functionality of allelic variations in human alcohol dehydrogenase gene family: assessment of a functional window for protection against alcoholism. Pharmacogenet Genomics.

[63] Kiage JN, James LO, Kabagambe EK (2014). Genetic modification of the effects of alcohol on metabolic and clinical phenotypes: a review. Curr Nutr Rep.

[64] Kang G, Bae KY, Kim SW, Kim J, Shin HY, Kim JM, Shin IS, Yoon JS, Kim JK (2014). Effect of the allelic variant of alcohol dehydrogenase ADH1B* 2 on ethanol metabolism. Alcohol Clin Exp Res.

[65] Ferrari P, McKay J, Jenab M, Brennan P, Canzian F, Vogel U, Tjønneland A, Overvad K, Tolstrup JS, Boutron-Ruault M (2012). Alcohol dehydrogenase and aldehyde dehydrogenase gene polymorphisms, alcohol intake and the risk of colorectal cancer in the European Prospective Investigation into Cancer and Nutrition study. Eur J Clin Nutr.

[66] He L, Deng T, Luo H-S (2015). Genetic polymorphism in alcohol dehydrogenase 2 (ADH2) gene and alcoholic liver cirrhosis risk. Int J Clin Exp.

[67] Hasin D, Aharonovich E, Liu X, Mamman Z, Matseoane K, Carr L, Li T-K. Alcohol and ADH2 in Israel: Ashkenazis, Sephardics, and recent Russian immigrants. Am J Psychiatry. 2014. 10.1176/appi.ajp.159.8.1432 12153842

[68] Ishiguro S, Sasazuki S, Inoue M, Kurahashi N, Iwasaki M, Tsugane S, Group JS (2009). Effect of alcohol consumption, cigarette smoking and flushing response on esophageal cancer risk: a population-based cohort study (JPHC study). Cancer Lett.

[69] Crous-Bou M, Rennert G, Cuadras D, Salazar R, Cordero D, Rennert HS, Lejbkowicz F, Kopelovich L, Lipkin SM, Gruber SB (2013). Polymorphisms in alcohol metabolism genes ADH1B and ALDH2, alcohol consumption and colorectal cancer.

[70] Ji YB, Tae K, Ahn TH, Lee SH, Kim KR, Park CW, Park BL, Shin HD (2011). ADH1B and ALDH2 polymorphisms and their associations with increased risk of squamous cell carcinoma of the head and neck in the Korean population. Oral Oncol.

[71] Liu S, Cui Y, Yang B, Chai P, Su Z, Zhang Q, Zheng D, Li R, Yu G (2015). Relationship between ADH2 Arg47His variation and hepatocellular carcinoma susceptibility: a meta analysis. Int J Clin Exp.

[72] Wang H-L, Zhou P-Y, Liu P, Zhang Y. ALDH2 and ADH1 genetic polymorphisms may contribute to the risk of gastric cancer: a meta-analysis. PLoS One. 2014;9. 10.1371/journal.pone.0088779 PMC3954547 24633362

[73] Chen YJ, Chen C, Wu DC, Lee CH, Wu CI, Lee JM, Goan YG, Huang SP, Lin CC, Li TC (2006). Interactive effects of lifetime alcohol consumption and alcohol and aldehyde dehydrogenase polymorphisms on esophageal cancer risks. Int J Cancer.

[74] Li D-P, Dandara C, Walther G, Parker MI (2008). Genetic polymorphisms of alcohol metabolising enzymes: their role in susceptibility to oesophageal cancer. Clin Chem Lab Med.

[75] Oze I, Matsuo K, Suzuki T, Kawase T, Watanabe M, Hiraki A, Ito H, Hosono S, Ozawa T, Hatooka S (2009). Impact of multiple alcohol dehydrogenase gene polymorphisms on risk of upper aerodigestive tract cancers in a Japanese population. Cancer Epidemiol Biomarkers Prev.

[76] Lee CH, Wu DC, Wu I, Goan YG, Lee JM, Chou SH, Chan TF, Huang HL, Hung YH, Huang MC (2009). Genetic modulation of ADH1B and ALDH2 polymorphisms with regard to alcohol and tobacco consumption for younger aged esophageal squamous cell carcinoma diagnosis. Int J Cancer.

